# Data on estimation for sodium absorption ratio: Using artificial neural network and multiple linear regressions

**DOI:** 10.1016/j.dib.2018.08.205

**Published:** 2018-09-05

**Authors:** Majid Radfard, Hamed Soleimani, Samira Nabavi, Bayram Hashemzadeh, Hesam Akbari, Hamed Akbari, Amir Adibzadeh

**Affiliations:** a Health Research Center, Life Style Institute, Baqiyatallah University of Medical Sciences, Tehran, Iran; b Department of Environmental Health, School of Public Health, Tehran University of Medical Sciences, Tehran, Iran; c Department of Environmental Health, School of Public Health, Khoy University of Medical Sciences, Khoy, Iran; d Research Center for Health Sciences, Institute of Health, Department of Environmental Health, School of Health, Shiraz University of Medical Sciences, Shiraz, Iran

**Keywords:** Groundwater quality, SAR, Aras, Neural network, Multiple linear regression

## Abstract

In this article the data of the groundwater quality of Aras catchment area were investigated for estimating the sodium absorption ratio (SAR) in the years 2010–2014. The artificial neural network (ANN) is defined as a system of processor elements, called neurons, which create a network by a set of weights. In the present data article, a 3-layer MLP neural network including a hidden layer, an input layer and an output layer had been designed. The number of neurons in the input and output layers of network was considered to be 4 and 1, respectively, due to having four input variables (including: pH, sulfate, chloride and electrical conductivity (EC)) and only one output variable (sodium absorption ratio). The impact of pH, sulfate, chloride and EC were estimated to be 11.34%, 72.22%, 94% and 91%, respectively. ANN and multiple linear regression methods were used to estimate the rate of sodium absorption ratio of groundwater resources of the Aras catchment area. The data of both methods were compared with the model׳s performance evaluation criteria, namely root mean square error (RMSE), mean absolute error (%) and correlation coefficient. The data showed that ANN is a helpful and exact tool for predicting the amount SAR in groundwater resources of Aras catchment area and these results are not comparable with the results of multiple linear regressions.

**Specifications table**

**Table:** 

Subject area	Chemistry
More specific subject area	Water quality and monitoring
Type of data	Tables, Figures
How data was acquired	Data on groundwater resources quality in the Aras catchment area was obtained from West Azerbaijan Water and Wastewater Company during the years 2010–2014 and was studied for estimation of sodium absorption ratio (SAR).
Data format	Raw, Analyzed
Experimental factors	The sodium absorption ratio (SAR), were analyzed according to the standards for water and wastewater treatment handbook.
Experimental features	The levels of physical and chemical parameters were determined.
Data source location	Aras, West Azerbaijan province, Iran.
Data accessibility	Data are included in this article
Related research article	A.Takdastana, M. Mirzabeygi (Radfard), M.Yousefi, A. Abbasnia, R. Khodadadia, A H.Mahvi, D.Jalili Naghan, Neuro-fuzzy inference system Prediction of stability indices and Sodium absorption ratio in Lordegan rural drinking water resources in west Iran, Data in Breif 18(2018)255–261.

**Value of the data**

• The data of this article can be used to environmental management and better exploitation of groundwater resources.
• Considering the present data, many of the sampling drinking water supply reservoirs need to pay attention to achieve Iran national water quality standards.
• The results clearly indicate that with appropriate selection of input variables, artificial neural network and multiple linear regressions as a soft computing approach can be used to estimate water quality indices properly and reliability.

## 1 Data

**Fig. 1:**
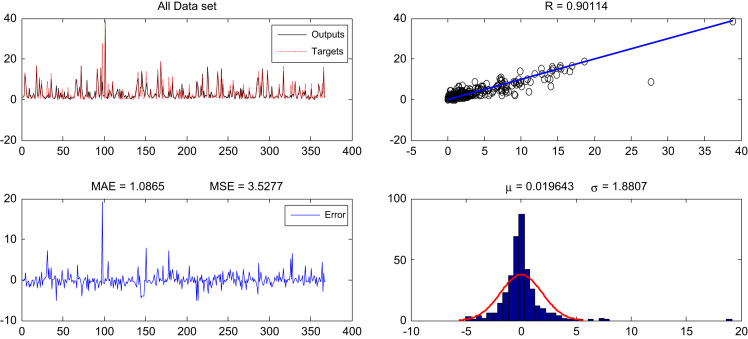
Optimized neural network output and model performance criteria for all data.

**Fig. 2:**
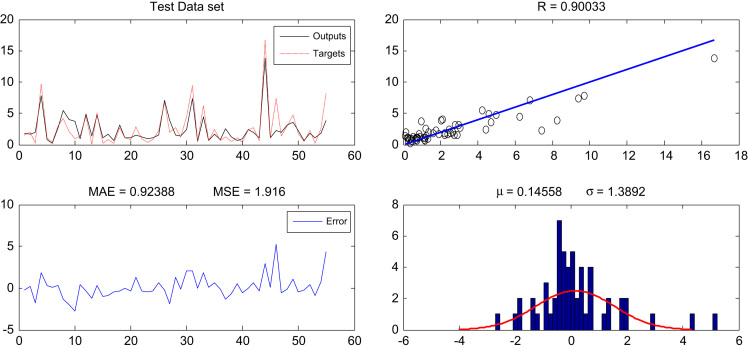
Optimized neural network output and model performance criteria for test data.

**Fig. 3:**
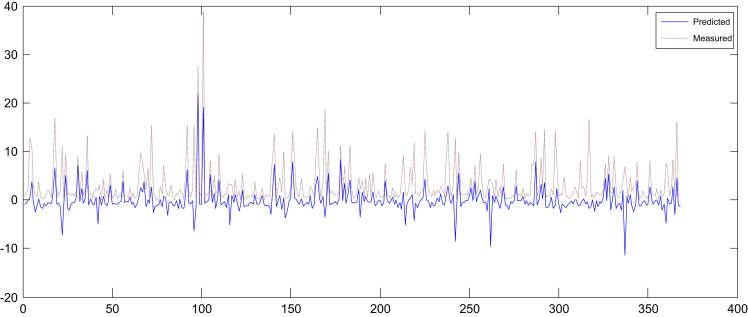
Actual SAR values in groundwater resources and their predicted values with multiple linear regression.

Two algorithms, including seven Back-propagation algorithms and Lewenberg-Markow, have been used in this data article. The Comparison of the performance of seven Back-propagation algorithms in estimating the sodium absorption ratio with the number of neurons 10 in the hidden layer has been shown in Tables 1 and 2, indicating the comparison of the different neurons performance in the hidden layer in estimating the sodium absorption ratio using the Lewenberg-Markow algorithm. The optimized output of neural network and data performance criteria for has been shown in Fig. 1, Fig. 2. Also, Fig. 3 indicates the actual SAR values in groundwater resources and their predicted values via multiple linear regression.

**Table 1:** Comparison of the performance of seven Back-propagation algorithms in estimating the sodium absorption ratio with the number of neurons 10 in the hidden layer.

Back-propagation algorithms	Neural network process	Evaluation of model performance			Repeat number
		*R*	MAE	MSE	
Trainbfg (BFGS quasi-Newton)	Training	0.883	1.34	5	132
	Test	0.819	1.33	4.54	132
	Training+Test+Validation	0.866	1.36	4.7	132
Traincgp (Polak–Ribie´re conjugate gradient)	Training	0.916	1.15	3.07	39
	Test	0.855	1.2	3.16	39
	Training+Test+Validation	0.893	1.2	3.78	39
Traingd (gradient descent)	Training	0.726	13.09	197.07	52
	Test	0.736	13.89	221.88	52
	Training+Test+Validation	0.762	13.17	193.17	52
Traingda (adaptive learning rate back-propagation)	Training	0.806	1.71	7.25	500
	Test	0.85	1.54	4.58	500
	Training+Test+Validation	0.811	1.68	6.55	500
Trainscg (scaled conjugate gradient)	Training	0.898	1.23	4.13	143
	Test	0.86	1.16	2.55	143
	Training+Test+Validation	0.89	1.25	3.85	143
Traincgf (Fletcher–Powell conjugate gradient)	Training	0.868	1.4	4.9	34
	Test	0.0804	1.52	4.49	34
	Training+Test+Validation	0.85	1.43	5.16	34
Trainlm (Levenberg-Marquardt)	Training	0.906	1.05	2.77	29
	Test	0.9	0.92	1.9	29
	Training+Test+Validation	0.0901	1.08	3.52	29

**Table 2:** Comparison of the different neurons performance in the hidden layer in estimating the sodium absorption ratio using the Lewenberg-Markow algorithm.

**Number of neurons**	***R***	**MAE**	**MSE**	**Repeat number**
3	0.879	1.23	4.59	25
5	0.86	1.24	4.81	32
7	0.91	1.22	3.61	25
10	0.9	0.923	1.9	29
12	0.89	1.14	4.03	33
17	0.88	1.09	2.37	29
20	0.895	1.08	3.76	30
30	0.892	1.09	3.92	34

## 2 Experimental design, materials and methods

### 2.1 Study area description

**Fig. 4:**
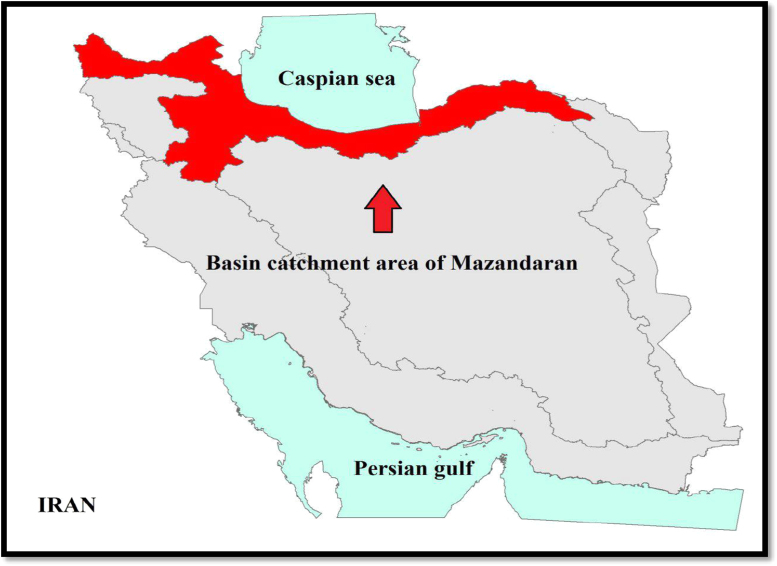
Study area.

Aras Catchment area is a plain located in the northern half of the East Azerbaijan province, West Azerbaijan province and Ardabil province. Extensive precipitation in this region, in addition to its impact on climate moderation, has created numerous rivers [18]. (Fig. 4).

### 2.2 Material and methods

Data on groundwater resources were collected a during the years 2010–2014 and water samples were analyzed following the standard methods for examination of water and waste water [1], [2], [3], [4], [5], [6], [7], [8], [9], [10] in terms of estimation of sodium absorption ratio (SAR). A two-layer neural network with a tangent-sigmoid transfer function for the hidden layer and a linear transfer function for the output layer was used. The input parameters of the neural network included sulfate, chloride, electrical conductivity (EC) and pH, and the sodium absorption ratio (SAR) were considered as the network output parameter. The data on these parameters were divided into training, testing and data validation. 70% of these data was used for training, 15% of data for validation and other 15% for testing. Considering that today BP neural networks have become a common tool for modeling environmental systems, so in this study, 8 BP algorithms were selected and their results were tested to obtain the best algorithm. For all algorithms, a dual layer network with a tan-sigmoid transfer function on the hidden layer and a linear transfer function in the output layer was used. In choosing the best BP algorithm, the number of neurons was considered 10. The results of the model׳s performance with their BP algorithm are presented in Table 1. The performance of the BP algorithm was evaluated with mean squared error (MSE), mean absolute error (MAE) and correlation coefficient (*R*) between the output of the models and the actual data set. The algorithm with the least training error and the maximum correlation coefficient was selected as the most suitable algorithm. The Langenberg-Marquard algorithm (trainlm) was chosen as the best algorithm to predict the sodium adsorption ratio (SAR). To optimize the number of neurons after selecting the best BP algorithm, namely Levenberg-Marquard, the number of neurons was optimized by keeping other parameters intact. As shown in Table 2, in the number of neurons greater than the optimal number of neurons, 10, the mean square error (MSE) was not significantly altered. Therefore, all the modeling steps were done based on the number of neurons 10 and the Lewenberg-Markow algorithm to predict the sodium absorption ratio [1], [2], [3], [4], [5], [6], [7], [8], [9], [10], [11], [12], [13], [14], [15], [16], [17], [18], [19], [20].
